# Evaluation of Serum Biomarkers and Other Diagnostic Modalities for Early Diagnosis of Preeclampsia

**Published:** 2019-06

**Authors:** Angela Felicia Sunjaya, Anthony Paulo Sunjaya

**Affiliations:** Faculty of Medicine, Tarumanagara University, Jakarta, Indonesia

**Keywords:** Preeclampsia, Early Diagnosis, Biomarkers, Doppler Ultrasonography, Diagnostic Model

## Abstract

**Objective:** Preeclampsia (PE) is a multi-systemic complication of pregnancy often characterised with the onset of hypertension and proteinuria after 20 weeks of gestation. Today, PE is the leading cause of maternal and perinatal morbidity and mortality worldwide. An early detection of PE would allow a chance to plan the appropriate monitoring and for clinical management to be immediately done following early detection thus making prophylactic strategies much more effective.

**Materials and methods:** This systematic review aims to evaluate the potential of the various serum biomarkers and diagnostic modalities (uterine artery Doppler, MAP, and maternal history) available for early prediction of PE with articles included and obtained through MEDLINE Full Text, Pubmed, Science Direct, ProQuest, SAGE, Taylor and Francis Online, Google Scholar, HighWire and Elsevier ClinicalKey.

**Results:** Ninety-five articles were found that fulfilled all of our inclusion criteria. Placental growth factor (PlGF), pregnancy associated plasma protein A (PAPP-A), soluble fms-like tyrosine kinase (sFLT) and placental protein 13 (PP-13) were the most commonly studied biomarkers. Whereas uterine Doppler scanning and Mean Arterial Pressure (MAP) were the most commonly studied out of other modalities.

**Conclusion:** Current evidence shows serum biomarkers such as PIGF, PP-13 and sFlt yielded the best results for a single biomarker with others having conflicting results. However, a combination model with other diagnostic modalities performed better than a single biomarker. In the future, new techniques will hopefully provide sets of multiple markers, which will lead to a screening program with clinically relevant performance. However further studies are required to improve current methods.

## Introduction

Preeclampsia (PE) is a multi-systemic complication of pregnancy often characterised with the onset of hypertension and proteinuria after 20 weeks of gestation, in a previously normotensive women (1, 2). On a global scale, PE is responsible for 14% of all maternal deaths and over 500,000 fetal deaths per annum making it the leading cause of maternal and perinatal morbidity and mortality (3, 4). Although its presentation is predominantly late term with a mild clinical course, severe maternal complications do occur most often resulting in marked elevations of blood pressure and end-organ dysfunction(5).

Severe PE can cause renal failure; hemolysis, elevated liver enzymes, and low platelet (HELLP) syndrome; liver haemorrhage and rupture; eclampsia; cerebral haemorrhage; and maternal death. Women suffering the condition has been found to carry a higher risk of complications and death resulting from their pregnancy with 10-25% of all PE cases resulting in maternal death and 15-20% of them resulting in preterm births (6-8). In addition to this, women with a history of PE have an elevated risk of cardiovascular diseases later in life (9, 10).

The impact of PE far extends from its mother to also its children. In foetuses, approximately 12-25% of fetal growth restriction (FGR) are attributable to PE (6). Newborns diagnosed with FGR at birth have a two to eight fold increased risk for hypertension, cardiovascular disease, diabetes mellitus or renal disease as adults (11, 12). They are also at risk of hypoxic induced neurologic injury and preterm delivery (2, 13). One quarter of still births and neonatal deaths are associated with PE and are especially prevalent in developing countries due to the lack of neonatal care facilities (6, 8).

Considering the importance of PE, the development of an efficient management system for the disorder is crucial to tackle the problem of PE. However, currently the diagnosis, screening and management of PE remains controversial and no single standard has so far been agreed upon (1, 14). The existence of a screening process would be essential to improve both pregnancy outcome and optimise the utilisation of resources in antenatal care (5). An early detection of PE would allow a chance to plan the appropriate monitoring and for clinical management to be immediately done following early detection of the disease thus making prophylactic strategies much more effective (13, 15). This systematic review aims to evaluate the potential of the various serum biomarkers and diagnostic modalities available for early prediction of PE.

## Materials and methods

The databases searched to obtain the articles included MEDLINE Full Text, Pubmed, Science Direct, Pro Quest, SAGE, Taylor and Francis Online, Google Scholar, High Wire and Elsevier Clinical Key. The search strategies used included availability of full text written in English from 1 January 2005 till 1 March 2018.

Keywords used were “early detection or synonyms” AND “first trimester or synonyms” AND “preeclampsia”. When multiple articles for a single study was found, the most recent publication was used. Relevance of studies was assessed by using an approach based on title, abstract and full text. Studies were included if they were original studies and if they have a Randomized Controlled Trial (RCT) or Cohort study design.

## Results

Our initial search resulted in 4,518 papers found, of which 4,280 were excluded on the basis of title and abstract. Of the remaining 238, 143 were excluded because they did not meet the inclusion criteria or were duplicated publications. Finally 95 articles were found that fulfilled all of our inclusion criteria.

Placental growth factor (PlGF), pregnancy associated plasma protein A (PAPP-A), soluble fms-like tyrosine kinase (sFLT) and placental protein 13 (PP-13) were the most commonly studied biomarkers. Whereas uterine Doppler scanning and Mean Arterial Pressure (MAP) were the most commonly studied out of other modalities. Outcomes of interest were the sensitivity, specificity and predictive value of modalities on predicting PE occurrence, reports on its advantages and drawbacks and its overall effect on mortality as well as morbidity of participants. It must be noted that studies included were based on published reports and take into account that studies reporting positive effects are more readily accepted to be published as compared to studies reporting negative effects.

## Discussion

Preeclampsia can be classified into early and late onset and is widely accepted as different forms of the disease. Early onset PE (EO-PE), requiring delivery 34 weeks before gestation, is commonly associated with adverse maternal and neonatal outcomes (6, 13). In contrast, late-onset PE (LO-PE), with delivery at or after 34 weeks, is mostly associated with mild maternal disease and a low rate of fetal involvement. The perinatal outcomes of late-onset PE are usually favourable (16, 17).

**Figure 1:**
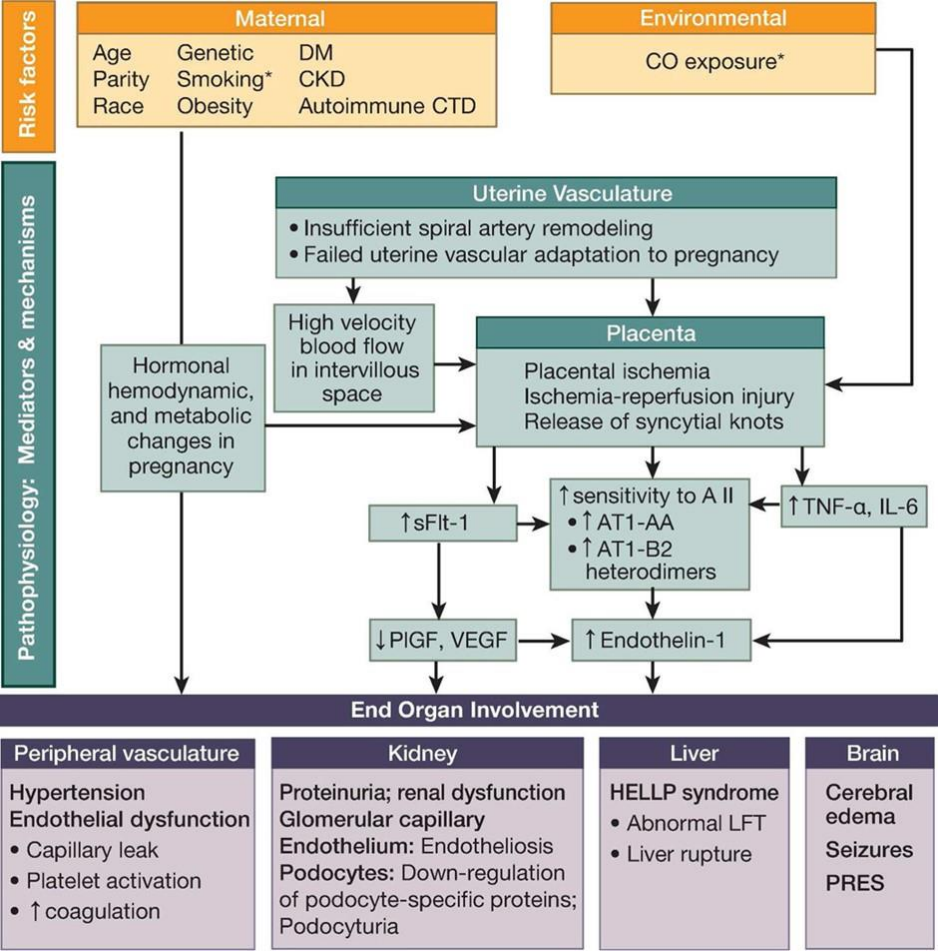
Etiology and Pathophysiologic Mechanisms of PE21

PE is considered to be a complex interaction between placental factors, maternal constitutional factors and immunological as well as vascular changes due to pregnancy resulting in the characteristic hypertension and proteinuria. Several pathophysiological mechanisms have been implicated in the pathogenesis of PE, however currently the mechanisms involved behind this disease still remains incompletely elucidated which complicates the prediction and treatment of PE (18-2) (Figure 1).

Currently, the diagnosis of PE is made on the basis of hypertension (arterial pressure exceeding 140/90 mmHg on at least 2 occasions, > 4 hours apart) and proteinuria (> 300 mg/dL/24h) found over 20 weeks of pregnancy (22, 23).

AT1-AA – autoantibodies to angiotensin II type 1 receptor; AT1-AA-B2 – heterodimers, angiotensin II type 1 receptor-bradykinin type 2 receptor heterodimers; CO – carbonmonoxide; CKD – chronic renal disease; CTD – connective tissue disease; DM – diabetes mellitus; HELLP – hemolysis, elevated liver enzymes, low platelet count; IL-6 – interleukin 6; LFT – liver function tests; PIGF- placental growth factor; PRES – posterior reversible encephalopathy syndrome; sFlt-1 – soluble fms-like tyrosine kinase 1; TNFα – tumor necrosis factor α; VEGF – vascular endothelial growth factor.

*Reduced risk of pre-eclampsia

In contrast, it is recommended by the World Health Organization and supported by recent meta-analyses that the prevalence of PE can potentially be halved by the initiation of low-dose aspirin at 16 weeks or earlier (1, 24-26).

No screening procedure to predict PE is recommended by the American College of Obstetricians and Gynecologists (ACOG) beyond obtaining an appropriate medical history to evaluate for risk factors which makes the early diagnosis of more than half of PE cases which occurs among healthy nulliparous women to be difficult (27, 28).

Extensive research in the last several years on these mediators and signalling pathways as a result of the need for early detection in the first trimester to reduce maternal mortality and morbidity has however identified a series of early biophysical and biochemical markers of PE (29, 30) (Table 1).

***Serum Biomarkers***

Due to the extensive number of biomarkers as shown in the table above, biomarkers were only evaluated if they have at least 10 journal articles discussing their diagnostic use.

*Vascular Endothelial Growth Factor and Placental Growth Factor (PlGF): *PIGF, a dimeric glycoprotein, is a member of the VEGF Family synthesized in villous and extravillouscytotrophoblast and has both angiogenicand pro-inflammatory properties (46, 47). PE occurs when impaired placentation followed by ischemia results in increasing circulation of anti-angiogenic factors (sFlt-1 and sEng) which later antagonize a number of pro-angiogenic factors such as PIGF (48, 49). As a consequence, the concentration of important angiogenic and PIGFs in maternal circulation is reduced evidently during the first and second trimester leading to impaired endothelial function and subsequently EO-PE (5, 50).

**Table 1:** Proposed maternal biochemical markers for the prediction of PE (31-46)

A disintegrin and metalloprotease 12 (ADAM12)	L-Arginine
Activin-A	L-Homoarginine
Adiponectin	Leptin
Adrcnomedullin	Magnesium
Alpha fetoprotein	Matrix mctalloproteinase-9 Microalbuminuria
Alpha-l-microglobulin	Microtransferrinuria
Ang-2 angiopoietin-2	N-Acetyl-/J-glucosaminidase
Antiphospholipid antibodies	Neurokinin B
Antithrombin III	Neuropeptide Y
Atrial natriuretic peptide	Neutrophil gelatinase-associated lipocalin
Beta2-microglobulin	P-Selectin
C-reactive protein	Pentraxin 3
Calcium	Placenta growth factor
Cellular adhesion molecules	Placental protein 13
Circulating trophoblast	Plasminogen activator inhibitor-2
Corticotropin release hormone	Platelet activation
Cytokines	Platelet count
Dimethylarginine (ADMA)	Pregnancy associated plasma protein-A
Endothelin	Prostacyclin
Estriol	Relaxin
Ferritin	Resistin
Fetal DNA	Serum lipids
Fetal RNA	Soluble cndoglin
Free fetalhemoglobin	Soluble fms-like tyrosine kinase
Fibronectin	Thromboxane
Genetic markers	Thyroid function
Haptoglobin	Total proteins
Hematocrit	Transferrin
Homocysteine	Tumor necrosis factor receptor-1 Uric acid
Human chorionic gonadotropin	Urinary calcium to creatinine ratio
Human placental growth hormone	Urinary kallikrein
Inhibin A	Vascular endothelial growth factor
Insulin-like growth factor	Visfatin
Insulin-like growth factor binding protein Insulin	Vitamin D
resistance	
Isoprostanes	

AT1-AA – autoantibodies to angiotensin II type 1 receptor; AT1-AA-B2 – heterodimers, angiotensin II type 1 receptor-bradykinin type 2 receptor heterodimers; CO – carbonmonoxide; CKD – chronic renal disease; CTD – connective tissue disease; DM – diabetes mellitus; HELLP – hemolysis, elevated liver enzymes, low platelet count; IL-6 – interleukin 6; LFT – liver function tests; PIGF- placental growth factor; PRES – posterior reversible encephalopathy syndrome; sFlt-1 – soluble fms-like tyrosine kinase 1; TNFα – tumor necrosis factor α; VEGF – vascular endothelial growth factor.

*Reduced risk of pre-eclampsia

Maternal serum levels of PIGF in pregnancies destined to develop PE, especially EO-PE, increases less or remains low throughout pregnancy.(51-53) Significant differences can be seen as early as 9 weeks, where PIGF can first be measured.(54) The detection rate (DR) of PIGF during the first trimester was found to be between 41% to 59% for EO-PE and 33% for LO-PE (51-56). This can be improved by the addition of serum PAPP-A and maternal history from 51% to 74% with a false positive rate (FPR) of 10% foe EO-PE and 33% to 43% with a false positive rate of 5% for LO-PE (55, 57).

*PAPP-A (Pregnancy Associated Plasma Protein A): *PAPP-A is a syncytiotrophoblast derived insulin-like growth factor binding protein protease and has long been used in risk calculation for chromosomal abnormalities such as Down’s syndrome. It regulates the bioavailability of free IGF at the placental-decidual interface during human implantation. Low concentrations of PAPP-A in the first trimester of pregnancy are highly associated with chromosomal aneuplodies. However, in chromosomally normal pregnancies, there is evidence that low maternal serum PAPP-A is linked with increased risk for subsequent development of PE (58).

The majority of studies found a significant association between PAPP-A and EO-PE with detection rates from 22% to 43%. However, it is not an effective standalone screening tool for PE because only 8-23% of affected cases have serum levels below the 5th percentile with the reported odds ratio varying between 1.5 and 4.6 (51, 55, 56, 59).

*Placental Protein 13 (PP-13): *PP-13 is a 32-kDa dimer protein belonging to the galectin super-family, a family of carbohydrate binding proteins called b-galactoside-specific lectins highly expressed in the placenta, specifically by the syncytiotrophoblast (60). PP-13 is suggested to be involved in the remodelling of the common fetomaternal blood-spaces through binding to proteins between the placenta and the endometrium. In normal pregnancies, serum PP-13 gradually increases to double or triple their values before delivery (61, 62). Several studies have demonstrated that low concentrations of PP-13 as early as 5-7 weeks predicts the onset of PE (41, 63). A number of studies on PP-13 found DR varying from 36% to 80% while Chafetz et al reported a quite high sensitivity (79%) and specificity (90%) ( 39, 54, 64, 65). These results can be further improved by combining PP-13 with maternal history and MAP as demonstrated by Gonen et al. with DR reaching 92% at a 12% FPR (41).

*Serum Soluble Flt-1: *sFlt-1 is a truncated splice variant of the membrane bound Flt-1. This splice variant circulates freely in the serum, where it binds and neutralizes VEGF and PIGF, preventing interaction with their biologically active transmembrane receptor (66). Several studies have reported sFlt-1 levels to rise as early as 5 weeks before the onset of PE. sFlt-1 levels are correlated directly with severity of disease and inversely related with the onset of clinical symptoms mainly hypertension and proteinuria (13,17, 67). sFlt-1 use however, would be improved if used in conjunction with other serum biomarkers such as PIGF instead (sFlt/PIGF ratio) as sFlt has a drawback of low sensitivity despite its high specificity (68). A study by Boucoiran et al, found sFlt to have a 90% specificity and 25% sensitivity (69). These findings are further supported by Myatt et al, with an 80% specificity and 21% sensitivity as well as various other studies (17, 35, 37, 70, 71).

*β-humanChorionic Gonadotropin (β-hCG): *β-hCG is a promoter of cell growth and differentiation in the embryo secreted by syncytiotrophoblastic cells of the placenta with the primary function of maintaining the vascular supply of placenta during pregnancy (72,73). In normal pregnancies, its levels increase until 9 to 10 weeks, decreasing afterwards. However, studies have shown that in those who develop PE, β-hCG levels continue to be elevated well beyond to the second trimester (5). Unfortunately, studies reviewed showed that it has a low predictive value for PE (34, 74-78).

However, a large scale retrospective analysis study by Cohen et al. involving almost 2,200 women showed that a combination of first and second trimester serum biomarkers, pregnancy-associated plasma protein A (PAPP-A), free βhCG, and maternal serum alpha-fetoprotein (msAFP) has a predictive probability of 91% of PE (73).

*Inhibin-A and Activin-A: *Inhibin-A and Activin-A are glycoprotein hormones produced by the fetoplacental unit which has been suggested to be involved in the feedback loop regulating hCG levels during pregnancy (5). An early study by Muttukrishna et al. showed that those suffering from PE has serum concentrations of both markers 10-times greater compared to controls in their third trimester (79).

Later on, several studies has exhibited that both inhibin-A and activin-A are increased in the maternal blood of first trimester patients who later develop PE compared to pregnant women with normal pregnancies (74, 78, 80-82). However, a study by Spencer et al. reported that when used alone, DR of inhibin-A and activin-A for PE to be 35% and 20% respectively. Better results were obtained when used with a combination of other markers (82).

*Soluble Endoglin: *Soluble endoglin is an auxiliary co-receptor of transforming growth factor β2 (TGF-β2). This co-receptor interferes with binding of TGF-β1 to its receptor, and thus was reported to play a role in the alterations in vasculogenesis and angiogenesis which occurs during PE, including effects on the production of nitric oxide, vasodilation and, capillary formation by endothelial cells as well as hypoxia and oxidative stress (83, 84).

Animal models have shown that sEng, interacts with another anti-angiogenic protein, sFlt-1 inducing severe PE-like disease (85). Various clinical studies has also reported that high levels of soluble endoglin was found in the plasma of women with PE.(85, 93) This was in contrast to 2 studies by Myers et al (35). And Myatt et al (37). Which reported no change in sEng levels.

However, a large scale prospective cohort study by Kusanovic et al. involving over 1,600 pregnant women studying sEng levels at early and mid-trimester concluded that it is a promising biomarker in predicting PE, although best diagnostic performances were obtained when combined with other biomarkers (42).

*A Disintegrin and Metalloprotease 12 (ADAM-12): *ADAM12 is a placenta-derived member of the ADAM protein family, hypothesised to take part in placental growth and development.(5) Furthermore, Gack et al. demonstrated that ADAM12 was the most upregulated transcription factor in placental tissues of women with PE (94). Several studies have examined ADAM12 serum levels between 8 to 14 weeks of pregnancy with conflicting results (74, 95-98). Reduced ADAM12 levels was reported by Laigaard et al. and Spencer et al. in pregnancies complicated by PE, both studies concluded the potential for PE as an early biomarker (96, 97). In contrast, studies by Poon et al, Audibert et al and Wortelboer et al. reported no change in ADAM12 levels in those suffering from PE (74, 95, 98). Further studies would thus be required to confirm these findings.

***Other proposed modalities***

*Maternal History: *Maternal history including ethnic origin, parity, body mass index (BMI) and personal or family history (PE, anti-phospolipid syndrome, chronic kidney disease, insulin-dependent diabetes, multiple pregnancies, pre-existing hypertension, and nulliparity) are well-known risk factors of PE (107, 108). Among women considered as high-risk, approximately 25% will develop PE compared with 5% in the general population (27) (Table 2).

**Table 2:** Other proposed maternal variables for the prediction of PE (29, 32-34, 37, 38, 40, 59, 74, 99-106)

**Parameter**	**Classified by category**	**Classified by risk profile**
Maternal age	Personal history	Personal risk profile
Maternal ethnicity		
Tobacco use in pregnancy		
Parity		
Education level		
Conception method		
Family history of preeclampsia		
Prior preeclampsia		
Prior preterm birth		
Renal disease	Past medical conditions	Cardiovascular risk profile
Hypertension		
Systolic blood pressure	Physical examination	
Diastolic blood pressure		
Mean arterial blood pressure		
Prior gestational diabetes	History	Metabolic risk profile
Pre-existing diabetes mellitus	Past medical conditions	
Maternal height	Physical examination	
Maternal weight		
Maternal body mass index		
Thrombophilia	Past medical conditions	Prothrombotic risk profile
Uterine artery Doppler index	Placental blood flow	
Uterine artery notching		

However screening strategies using maternal history alone for detection of PE perform moderately well at best. The National Institute of Clinical Excellence (NICE) found that a screening strategy based on maternal history and risk factors categorizes more than 60% of pregnant women as high risk and predicts fewer than 30% of those to develop PE with a false positive rate of 10%b (15, 109). As the pathophysiology of PE is thought to include abnormal placentation, the evaluation of uterine artery blood flow resistance may be able to support the results of risk factors (13). An assessment of risk by Poon et al. based on maternal history, blood pressure and uterine artery Doppler has found the detection rate of PE to be higher than based on maternal history alone (107, 109).

*Biophysical: *Biophysical modalities reviewed include blood pressure, mean arterial pressure (MAP) monitoring and uterine artery doppler scan (UtAD).

***Blood Pressure & Mean Arterial Pressure (MAP)***

Women who subsequently developed PE are found to have a higher systolic blood pressure and MAP readings before the onset of the disease – with MAP (the sum of systolic and twice the diastolic pressure divided by three) having a higher predictive value for PE among low risk women in the first and second trimester than either systolic or diastolic readings alone (13, 110, 111). Kumar et al discovered that a combination of mean BMI, MAP, uterine artery Doppler and pulsatility index, have a distinct correlation with the onset of hypertension with a sensitivity and specificity of 76% and 80% respectively (112).

First-trimester MAP has been shown to be affected by maternal weight, height, age, racial origin, cigarette smoking, family and prior history of PE, and history of chronic hypertension. Consequently, combining these predictors may be beneficial in selecting individuals for close monitoring and early intervention during pregnancy (29). In a study by Poon et al, a combination of MAP with maternal risk factors resulted in a detection rate of 76% (113). A study by Gallo et al. performed MAP-1 (during 11-13 weeks) and MAP-2 (20-24 weeks) screening, which showed detection rates of 74.3% and 84.3% for EO-PE respectively thus proving that MAP would be best taken during these weeks (114, 115).

Maternal MAP is an easy, cost effective, and non-invasive test that can be performed to all women during their first antenatal visit. MAP can also be combined with uterine artery Doppler and biomarkers where a study reported this combined screening, with a false positive rate of 5% identified approximately 80% of PE patients before 34 weeks gestation and another study reported that 90% of PE developing pregnancies were identified in the subsequent 4 weeks (46, 55, and 57).

***Uterine Artery Doppler (UtAD)***

The impedance to blood flow in the uterine arteries steadily declines in the progression of a normal pregnancy where this transformation is necessary to ensure a dramatic increase of blood supply to intervillous space (46, 63, 116). However due to defective differentiation of trophoblast and impaired invasion of spiral arteries, the uteroplacental circulation remains in a state of high resistance which can be measured noninvasively by UtAD.(113, 117)UtAD uses color flow mapping to identify vessels before a pulsed wave Doppler is used where the three similar consecutive waveforms are used to obtain the Pulsatility Index (PI) and calculate the mean PI of the left and right arteries (118).

A feature of UtAD is that the detection rates and sensitivity are better suited for second trimester than first trimester screening and for pre-term/early form PE than for severe or mild PE (99, 115). A meta-analysis by Velauthar et al involving 11 studies (43,122 pregnancies) to predict PE in the first trimester with UtAD reported that abnormal UtAD has a high specificity (91%) and low sensitivity (26%) in the first trimester (119). Nevertheless, it must be kept in mind that first trimester UtAD are shown to be affected by maternal history and risk factors and adjustment must be taken (29). Considering this, a combination of maternal factors and UtAD has yielded a better detection rate (from 51% to 75%, with a false positive rate of 10%) for PE requiring delivery before 37 weeks gestation (46).

The use of UtAD remains controversial with its conflicting results however despite it, the evaluation of UtAD suggests it as an important predictor as it will allow clinicians to determine women at risk of especially EO-PE, which causes the highest perinatal morbidity and mortality, and IUGD, enabling preventive methods to be taken to minimise adverse outcomes (13, 120).

***Combination Studies***

*sFlt/PIGF Ratio: *The PIGF/sFlt-1 ratio has been brought forward as a possible predictive marker not only for preeclampsia butalso other placenta related disorders such as IUGR or stillbirth (2, 121). In a multicentre clinical study by Verlohren et al, it was reported that the PGF/sFlt-1 ratio has a DR of 82% with a 5% FPR and is especially high for EO-PE (89% detection rate). Other than these findings, it was especially important to know that PE patients with a high PIGF/sFlt-1 ratio were found to have a significantly increased risk for delivery within 7 days as the early identification and timely referral to a perinatal care unit is hypothesised to be able to reduce perinatal morbidity and mortality by 20% (122). Benton et al also performed a case control study of 44 patients to compare two commercially available sFlt/PLGF assay with both having greater than 95% sensitivity and a greater tendency for detection in the diagnosis of EO-P (123).

*PlGF, sEng, and soluble fms-like tyrosine kinase 1 (sFlt1) Ratio: *A large scale prospective cohort study by Kusanovic et al. involving over 1600 pregnant women studying sEng levels at early and mid-trimester concluded that best diagnostic performances were obtained by ratios in the mid-trimester and the slopes of plasma concentrations of PlGF, sEng, and soluble fms-like tyrosine kinase 1 (sFlt1) between the first and second trimester with a sensitivity of 100% for all tests and a specificity between 98-99% (42).

*Maternal Characteristics, Biophysical and Biochemical Markers: *A recent large scale study involving over 50,000 singleton mothers by Akolekar et al. developed a new model for PE screening wherein the combined use of maternal characteristics (race, method of conception, smoking, history of diabetes and hypertension, history of autoimmune disorders, family history of PE and obstetric history) with biophysical characters namely uterine artery pulsatility index and MAP together with serum biomarkers PAPP-A and PLGF was able to detect 96% of cases of early onset PE cases and 54% of all PE cases at a fixed false-positive rate of 10% (124).

Many other models have also been develop involving the combined use of various different maternal characteristics, biophysical characteristics and serum biomarkers with differing sensitivity and specificity (28, 31, 34, 35, 38, 41, 73, 74, 90, 101, 106, 107).

Further large scale studies comparing their uses with each other in diverse populations need to be done before these models can be implemented at large.

***Limitations of the Review***

This review article suffers limitations in only being able to synthesize journal articles and trials previously published which are accessible to the authors.

***Concerns and Practical Implications***

Although extensive research has been done focusing on the identification of women at high risk of developing PE, various studies have reported that PE cannot be predicted by previous obstetric history and risk factors alone (68, 106). PP-13 was found to be the best at predicting EO-PE as a single biomarker. However, no single marker was able to perform better compared to a combination model taking into account the various facets of PE.

As several large scale studies have shown, serum markers when combined with maternal characteristics such as age, weight, height, nulliparity, smoking status, first-trimester MAPand, uterine artery Doppler are powerful tools, able to predict PE in the first trimester. 

From a public health perspective, a cost effective solution can be found through the use of clinical, uterine artery Doppler and MAP assessments, negating the use of the costly laboratory based biomarker(s) prediction strategy. As the study by Kuc et al. has shown, maternal characteristics when combined with first-trimester MAP and UtAD was able to have a high detection rate of 89.2%, in line with other studies as well (32).This may seem plausible as maternal characteristics determine the susceptibility of a mother and MAP relates to maternal vascular adaptation. Whereas biomarkers relate to placentation.

No matter the modality chosen, early detection for PE especially EO-PE is one that we must strive for, to reduce both maternal and neonatal morbidity as well as mortality. Early prediction would allow for greater risk assessments and control, more intensive monitoring of this high risk group as well as targeted prophylactic intervention, timely diagnosis and treatment.

## Conclusion

PE is a hypertensive pregnancy disorder with multifactorial origins. First trimester screening for PE will allow for a personalized risk estimate to the population early in their pregnancy. Serum biomarkers such as PIGF and sFlt yielded the best results for a single biomarker with others having conflicting results. However, a combination model with other diagnostic modalities including maternal history, MAP, and uterine artery Doppler performed better than a single biomarker. Further research is needed as current models have a low predictive value and guidelines need to be created to implement this method in Indonesia. In the future, new techniques will hopefully provide sets of multiple markers, which will lead to a screening program with clinically relevant performance and early prophylactic strategies could be made for high risk women.
